# Utilization of By-Products from Livestock: Study on the Mechanical and Thermal Properties of Biodegradable Containers Made with Pork Skin Gelatin Polymer

**DOI:** 10.3390/foods11162513

**Published:** 2022-08-19

**Authors:** Sol-Hee Lee, Hack-Youn Kim

**Affiliations:** Department of Animal Resources Science, Kongju National University, Yesan 32439, Chungnam, Korea

**Keywords:** biodegradable container, gelatin polymer, walnut shell, by-products, sustainable goals

## Abstract

This study aimed to develop a biodegradable container made of pork gelatin. Gelatin was extracted from pork skin by hot water at 80 °C, and containers were prepared by adding eggshell powder (20%) as a pore agent, and walnut powder (0.08 wt%; PEW1, 0.14 wt%; PEW2) to improve hardness. The blends were molded for each experiment and dried at 30 °C for 24 h, at 40 °C for 16 h, and at 121 °C for 16 h. The containers were analyzed with respect to morphological (SEM; scanning electron microscope), mechanical (tensile strain and stress), and thermal (DSA; differential scanning calorimetry and TGA; thermogravimetric analysis) properties, as well as biodegradability. SEM investigation showed a smoother surface for PEW1 than for PEW2. The tensile stress of PEW2 (37.86 MPa) was significantly higher than that of PEW1 (28.40 MPa), and the melting enthalpies were 137.60 J/g (PEW1) and 309.60 J/g (PEW2). TGA showed similar properties, but PEW2 contained more lignin; therefore, its decomposition temperature was higher. The PEW1 and PEW2 containers were completely biodegraded after approximately 7 and 11 weeks, respectively. Walnut shell powder increased the hardness, but slowed the biodegradation process. The applications of this biodegradable container are short-lived products such as food packaging.

## 1. Introduction

Home meal replacement (HMR) refers to food that can be easily consumed, replacing meals prepared at home, and the global HMR market is on the rise [1]. According to Research and Markets [2], global ready-meal products are projected to grow at a compound annual growth rate of 6.83% from 2019 to 2025. Currently, as the proportion of people eating at home has increased owing to the COVID-19 pandemic, the use of plastic as a packaging material for HMR products has continued to increase [3]. As of 2020, approximately 9% of the 8.3 billion tonnes of plastics was recycled, 12% was incinerated, and the rest was mostly disposed of in landfills or as environmental waste [4]. Accordingly, the importance of eco-friendly containers is emerging worldwide, and their continuous development is in progress.

Eco-friendly packaging materials include biodegradable and biomass products. Biodegradability refers to decomposition by microbes when the material is buried in soil [5]. The material is converted into environmentally friendly carbon compounds, water and inorganic compounds by insects and microorganisms [6]. According to Zurier and Goddard [7], as biodegradable materials are decomposed, the possibility of improving the environment by biocatalysis can be expected. Biodegradable containers are relatively easy to remove compared to non-degradable containers, but they have the disadvantage that the storage period for food is shorter [8]. Therefore, it is necessary to study the manufacture of biodegradable and environmentally friendly containers with enhanced physical properties.

According to the research results of Yang and Park [9], collectivism, ecology, and health affect the consumption value of food, and ethical consumerism and food safety act as major variables. It is important that eco-friendly containers do not affect the preservation of food, but factors such as durability, low toxicity, recyclability, and biodegradation also play an important role [10]. In light of this consumer psychology, patents are being developed and studies on product development using plant by-products are being conducted in Korea [11,12]. Cellulose extracted from plant by-products is the most abundant polymer found in nature, and has been used as a major polymer in the packaging field until now [13]. Cellulose has also been processed and used in the form of hydroxypropyl-methylcellulose (HPMC) and carboxymethyl cellulose nitrate (CMCN). However, these materials have weak resistance to water absorption; therefore, a supplementary point is needed [14].

Gelatin is mainly obtained from pork skins (46%) and pork or cattle bones (23.1%), and is used industrially [15]. Gelatin used for commercial purposes is swollen and heated to be processed and used in various forms, and the chain shape changes depending on the process used: single chain, two single chains/single chain with a loop, and three single chains with one loop or two single chains [16]. Gelatin is found in 27 different forms depending on pH and temperature conditions [17], and has high adhesion when converted to a collagen state by hydrothermal treatment [18]. It has been reported that the strength of gelatin increases after gelation is completed, and the higher the purity of the gelatin, the higher the strength maintained [19]. Owing to these properties, gelatin is used not only as a gel emulsion, an adhesive, and in medicinal capsules, but also as a medical tissue adhesive [20,21].

By-products such as gelatin are substances that are produced during the manufacturing and processing of food. These by-products are thrown away, because they are difficult to ingest for humans or require processing before they can be used. Therefore, the consumed pork skin, eggshell and walnut shell were used as a polymer, a pore agent, and a flavoring agent, respectively, to prepare a container. As interest in fields requiring biodegradable substances increases, we developed a biodegradable container that can be used in the food industry. To find out whether this biodegradable container can be used in industry, we studied biodegradable containers’ morphology, physics and biodegradability properties using a pork gelatin polymer.

## 2. Materials and Methods

### 2.1. Material and Preparation of Pork Gelatin Powder

Pork skin purchased from local markets (Yesan, Chungnam, Korea) was used for the production of gelatin [22]. Excess fat and impurities were removed from the pork skin by washing with water. The prepared material was allowed to swell for 24 h by diluting by a factor of ten (*v*/*w*) using 0.1 N HCl (purity 35–37%, pH 2). The swollen sample was placed in flowing water and neutralized until the pH reached 5.5. The samples were vacuum packed and extracted in a water bath (JSWB-30T, JSR, Gongju, Korea) at 80 °C for 2 h. The extracted gelatin was dried with hot air in an atmospheric dryer (C-F03, Vision Scientific, Daejeon, Korea) set at 105 °C, and walnut powder and eggshell powder were purchased from Ganong Bio (Incheon, Korea) and The witch company (Seoul, Korea), respectively; the particle size was 180 mesh.

### 2.2. Manufacture of Biodegradable Containers

**Figure 1:**
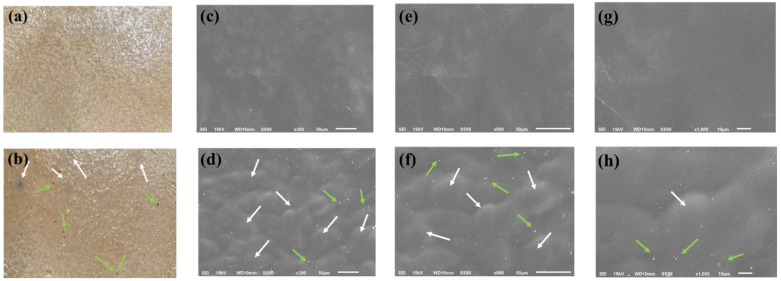
Images of biodegradable containers made from pork skin gelatin, walnut shells, and eggshells. (**a**) Surface of PEW1: pork gelatin, eggshell and 0.08 wt% walnut shell; (**b**) surface of PEW2: pork gelatin, eggshell and 0.14 wt% walnut shell; (**c**,**e**,**g**) representative SEM photographs of PEW1 surfaces at magnifications of 300, 500 and 1000×, respectively; (**d**,**f**,**h**) representative SEM photographs of PEW2 surfaces at magnifications of 300, 500 and 1000×, respectively.

Biodegradable containers were manufactured by mixing eggshell powder (purity 100%) and walnut powder (purity 100%) with the prepared gelatin [23] using the following method for establishing mixing ratios. The prepared gelatin powder was hydrated by adding three volumes of distilled water to a water bath (JSWB-30T, Korea) at 100 °C for 3 h. Then, 20% eggshell powder was added to the liquid gelatin. Two mixtures were prepared using this mixture: 0.08 wt% walnut powder (pork gelatin, eggshell and 0.08 wt% walnut shell; PEW1) and 0.14 wt% walnut powder (pork gelatin, eggshell and 0.14 wt% walnut shell: PEW2). The eggshell and walnut powder were stirred at 80 °C for 3 h until it was completely dissolved in the pig skin gelatin solution for every step. The final mixtures were prepared by stirring at 80 °C for 2 h in a water bath (JSWB-30T, Korea). The prepared mixture was poured into a 30 × 20 cm plate, cooled, and cut into smaller pieces for each experiment. The drying process was carried out at 30 °C for 24 h, at 40 °C for 16 h, and at 121 °C for 16 h in an atmospheric dryer (C-F03, Korea). The dried containers were sprayed with a 1% sodium alginate dilution solution and 1% calcium lactate for waterproofing [24], and dried at 30 °C for 24 h in an atmospheric dryer (C-F03, Korea). The biodegradable container is shown in Figure 1.

### 2.3. Morphological Properties

The mechanisms of surface fracture were determined using a Scanning electron microscope (SEM; ECLIPSE Ci-L, Nikon, Tokyo, Japan) and images were used to analyze the morphology of the samples. The SEM sample was prepared by cutting to 35 mm × 35 mm × 10 mm. The images were taken at a voltage of 15 kV and magnifications of 300, 500 and 1000×.

### 2.4. Mechanical Properties 

The samples for ultimate tensile stress and ultimate tensile strain were cut to 10 mm × 10 mm × 20 mm and 30 mm × 40 mm × 35 mm, respectively. The ultimate tensile stress and ultimate tensile strain of the samples were measured using a universal testing machine (AGS-X, Shimadzu Corporation, Kyoto, Japan) at a crosshead speed of 1 mm/min. The test conditions were 20 °C and 45% relative humidity. The tensile stress and strain were calculated based on the obtained tensile stress-strain curves. The experiments were repeated three or more times in total.

### 2.5. Thermal Properties

DSC and TGA were carried out on powder with a particle size of 32 mesh. DSC (differential scanning calorimetry) was used to measure the behavior of particles on a DSC-Q20 (TA Instruments, New Castle, DE, USA). The sample weight was 3–3.8 mg and analysis was carried out at a heating rate of 10 °C/min in the range of 0 to 250 °C. The experiment was conducted as follows: 1st heating → 1st cooling → 2nd heating → 2nd cooling. The indium (99.99%) was used for the temperature and enthalpy calibration in the experiment. Tg was measured as the midpoint of the transition during the scan, which was obtained by repeating the experiment twice. TGA (thermos-gravimetric analysis) was performed using TA-Q500 (TA Instruments, New Castle, DE, USA). Approximately 7–10 mg were weighed into a platinum pan and heated over the temperature range of 0 to 800 °C at a heating rate of 10 °C/min under a nitrogen purge [25].

### 2.6. Biodegradability Performed Indoors

To exclude variables, biodegradability was determined indoors in a simulated natural environment [26]. The soil was collected from the surface layer and placed in a 36 L plastic tray at a thickness of approximately 23 cm. After planting crops to create a natural environment, an analysis was performed. Uniform samples weighing 10 g were placed on the soil between plants where the roots of the crops were not in contact with the samples. A total of 4 or more samples were used per treatment group, and this was repeated twice. The test was performed for a total of 4 weeks, and the weight was checked every week. Water was sprayed once every four days to maintain moisture, and the decomposed sample was gently washed with distilled water and dried at 37 °C. After drying, the weight of each sample was determined. Based on the weight of the dried samples, the decomposition curve was plotted:

D_1_ [Biodegradability (%)] = (m_1_ − m_2_)/m_1_ × 100(%)

where m_1_ = amount of the injected test material (g), and m_2_ = amount of the recovered test material (g).

### 2.7. Statistical Analysis

The biodegradation curve was duplicated by performing at least three or more repeated experiments. Statistical analysis was performed using the statistical processing program SAS (Windows version 9.4, SAS Institute, Cary, NC, USA), and ANOVA, and Duncan’s multiple range test were used to determine if there was a significant difference for each characteristic.

## 3. Results

### 3.1. Scanning Electron Microscopy

SEM images of the outer surface of the biodegradable container made of pork skin gelatin polymers, eggshell, and walnut powder are shown in Figure 1. External observation of the biodegradable container with 10% walnut powder showed a smooth surface without any convex parts, but the biodegradable container with 20% walnut powder appeared rougher (green arrow). This phenomenon is likely due to the excess addition of walnut powder compared to the amount of gelatin. The black spots seen in Figure 1a,b appear to be a walnut powder (white arrow). In the SEM images, the presence of holes in the biodegradable surface is indicated by white dots, indicated by green arrows. This also appears in the biodegradable container with 20% walnut powder and seems to be due to the agglomeration of walnut powder. Katekhong et al. [27] reported that an inaccurate polymer blending ratio may lead to matrix breakdown. Therefore, it was decided that the addition of only 10% walnut powder was better for binding with gelatin.

### 3.2. Mechanical Properties

A universal testing machine (UTM) was used to analyze the mechanical properties of the materials. Analysis of the results can help in determining the best mixing ratio of materials and processing methods [28]. Table 1 shows the ultimate tensile stress (MPa) and ultimate tensile strain (%) of the biodegradable containers prepared with pork skin gelatin, eggshell and walnut powder. The tensile stress was 28.40 MPa in the container with 10% walnut powder (PEW1), significantly lower than that of the container with 20% walnut powder (PEW2) (37.86 MPa) (*p* < 0.05). Tensile strength and compressive are reported to have a positive correlation at an appropriate mixing ratio, and Nataraja and Dhang (2001) reported that compression tensile was 0.09 times of its compressive strength [29,30]. Although the tensile strain value of the PEW2 was higher than that of PEW1, the difference was found not to be significant (*p* > 0.05). Therefore, it can be concluded that PEW2, with a higher tensile strain, will have a higher compressive strength than PEW1. It was determined that the biodegradable container containing more walnut powder had a higher density. The addition of an excess of walnut powder to the polymer could interfere with the uniformity of the mixture [31]; however, no negative effects on uniformity were seen with the addition of 20%. Phothisarattana et al. [32] reported that solid particles in a polymer matrix can reinforce strength, and showed similar results to this study. It has been reported that tensile strength decreases as the particle size of agents used for biodegradable containers increases [33]. Therefore, if it is necessary to develop a container with high tensile strength, which may be achievable by reducing the amount of eggshell and walnut powder particles.

### 3.3. DSC (Differential Scanning Calorimetry)

**Figure 2:**
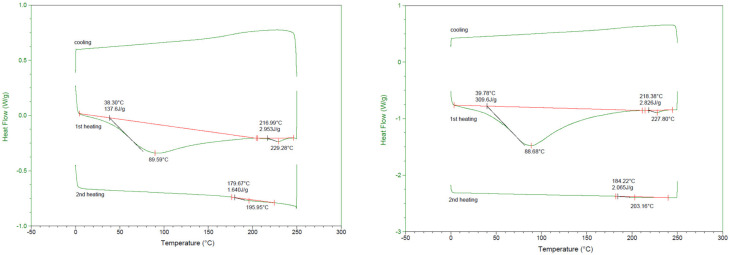
DSC scans of biodegradable containers made from pork skin gelatin, walnut shells, and eggshells.

The results of thermal analysis of the biodegradable containers made of gelatin polymer material are shown in Figure 2 and Table 2. There are several factors that affect DSC results, including polymer quality, heat capacity and thermal stability. In this study, DSC was conducted to analyze the characteristics of the unknown polymers [34]. Because both PEW1 and PEW2 show two transition points, the biodegradable containers made of gelatin, eggshell and walnut powder feature both a Tg (glass transition temperature) and a Tm (melting temperature), so it is considered a crystalline polymer. Xi Wang et al. [35] reported that the Tg is due to the Brownian motion of the amorphous polymer and Tm is the melting point of materials that appear only in crystalline polymers. The Tg values of PEW1 and PEW2 were 38.30 and 39.78, respectively, and the Tm values were 89.59 and 88.68, respectively. Rebers et al. [36] reported that the Tm value of gelatin was 28.0, which is much lower than in this study. This is because gelatin is a triple helical structured protein that exhibits the behavior of an upper critical solution temperature, and thus has high binding ability with other materials [37]. In contrast, the amount of change in the melting enthalpy of the sample during the heat scan was 137.60 J/g for PEW1 and 309.60 J/g for PEW2, indicating a large difference. This is considered to be due to the amount of hydrogel bonding in the helical structure of gelatin. Bigi et al. [38] reported different crosslinking according to the binding agents used for the gelatin polymer. In the 2nd heating, since the DSC of the mass excluding the mass removed in 1st was measured, it showed a lower value than in 1st. The PEW1 showed overall lower values of Tg, Tc and Hf compared to those of PEW2, this can be understood in the same context as the tensile strain (%), because PEW2 is a denser material.

### 3.4. TGA (Thermogravimetry Analysis)

**Figure 3:**
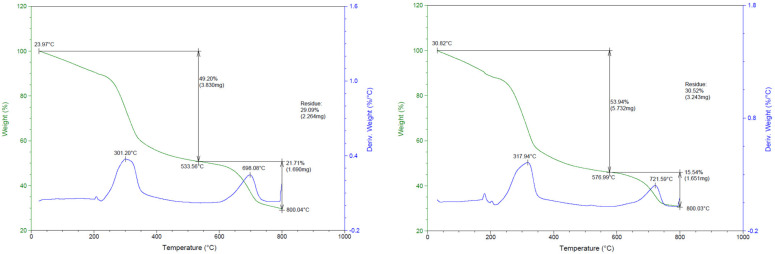
TGA scans of biodegradable containers made from pork skin gelatin, walnut shells, and eggshells.

TGA is not only a good indicator of the thermal stability of polymer materials, it is also a good method for measuring the decomposition temperature of biodegradable polymer materials [39]. Accordingly, Figure 3 shows the weight loss (%) curve and derivative weight (%/°C) plotted against the degradation temperature, with different amounts of walnut powder. In the TGA curves of both materials, three different weight losses are observed. The weight loss at 1–200 °C is considered to be due to the decomposition of the eggshell powder and the low molecular weight sodium alginate sprayed as a coating [40,41]. Thereafter, the weight of the PEW1 biodegradation container was reduced to 49.20% at 533.56 °C, and that of PEW2 was reduced to 53.94% at 576.99 °C, showing significant weight loss. This is similar to the decomposition temperature of lignin present in lignocellulosic biomass [42]. The two samples show the same result because they are similar materials, but PEW2 contains a higher amount of walnut powder per gram. In the third loss section, the two samples show similar final temperature values of 800.04 °C (PEW1) and 800.03 °C (PEW2). PEW1 shows initial decomposition at 225 °C and peak thermal decomposition at 301.20 °C. A second decomposition occurs at 620 °C, and the third decomposition at 696.08 °C. PEW2 shows double decomposition at 210 and 670 °C, and peak thermal decomposition at 317.94 and 721.59 °C. Overall, PEW1 and PEW2 show similar behavior and derivative weight, but it can be seen that PEW2 has slightly higher decomposition temperatures. This is thought to be because PEW2 contains more substances such as lignin, thus raising the decomposition temperature [43].

### 3.5. Biodegradability

**Figure 4:**
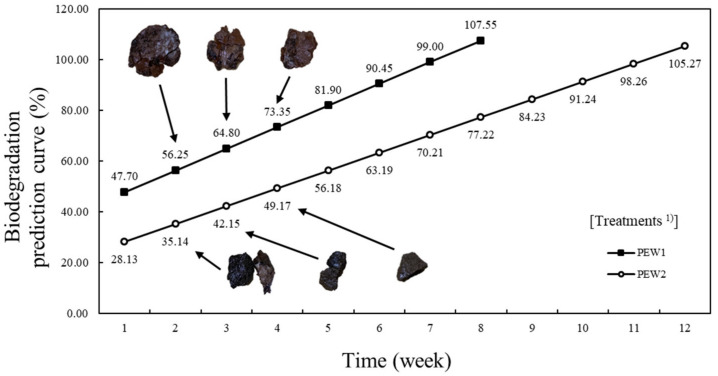
Biodegradation prediction curve (%) for biodegradable containers made from pork skin gelatin, walnut shells, and eggshells. ^1^ PEW1: pork gelatin, eggshell and 0.08 wt% walnut shell; PEW2: pork gelatin, eggshell and 0.14 wt% walnut shell.

The biodegradability of the containers made of gelatin polymer material was analyzed using a soil burial method similar to that of the external environment (Figure 4). Biodegradable packaging materials not only decompose naturally due to microorganisms in the soil and have no effect on the environment, they are also useful because they provide nutrients to the soil [44,45]. In this study, based on experimental results over up to four weeks, during which time the shape of the sample can still be distinguished, a prediction of the time at which 100% decomposition will occur is shown. Evidently, as decomposition was estimated by measuring the rate of decrease in weight over time, it cannot be completely excluded that damage to the sample had no effect [46,47]. Nevertheless, biodegradability can be inferred based on the results of this study. In the first week, PEW1 showed a degradation rate of 47.70%, and PEW2 showed a degradation rate of 28.13%. It was later confirmed that PEW1 was completely biodegraded at approximately 7 weeks and PEW2 was completely biodegraded at approximately 11 weeks; therefore, the rate of degradation of PEW1 was much faster (*p* < 0.05). Oyeoka et al. [48] reported that incompletely dispersed composite materials are easier to physically damage than completely dispersed composites. Therefore, it can be inferred from the experimental results that PEW2 has safer dispersion than PEW1. In addition, PEW1 had a larger surface area and decomposed faster than PEW2 (Figure 4). This result is attributed to the fact that PEW2 is a denser material. In addition, from the fifth week onwards, the amount of decomposition was difficult to measure. It is reported that the packaging material of food is affected by the sorption and diffusion between water and hydrophilic matrix [49]. The higher the hydrophilicity, the faster the biodegradation, therefore the actual decomposition rate may be faster than the predicted decomposition curve shown in Figure 4 [50].

## 4. Conclusions

With the utilization of by-products from livestock, the development of biodegradable containers made with pork gelatin showed the following results. Biodegradable containers with less walnut powder (PEW1) had a smoother appearance than PEW2. Additionally, PEW1 had a shorter biodegradation time compared to PEW2. Biodegradable containers with more walnut shell powder (PEW2) had higher tensile strain, stress and melting enthalpy. PEW1 could be useful in consideration of its clean appearance and biodegradation period, but when product strength is required, PEW2 showed better results. Therefore, it seems necessary to select the amount of walnut powder in biodegradable containers made of gelatin, varying its value according to the use, thickness and shape of the product being stored in such a container.

## Figures and Tables

**Table 1:** Mechanical properties of biodegradable containers made from pork skin gelatin, walnut shells, and eggshells.

Treatments ^1^	Ultimate Tensile Stress (MPa)	Ultimate Tensile Strain (%)
PEW1	28.40 ± 0.72 ^b^	26.23 ± 0.68 ^NS^
PEW2	37.86 ± 1.38 ^a^	33.37 ± 1.26

^1^ PEW1: pork gelatin, eggshell and 0.08 wt% walnut shell; PEW2: pork gelatin, eggshell and 0.14 wt% walnut shell. All values are mean ± SE. ^a,b^ Means in the same column with different letters are significantly different (*p* < 0.05). ^NS^: non-significant.

**Table 2:** Analysis of the effect of seal integrity of DSC sample pan on thermal transitions of biodegradable container of pork skin gelatin, walnut shells, and eggshells.

Treatments ^1^	Sample mass/mg	1st Heating			2nd Heating		
		T_g_/°C	T_m_/°C	ΔH/J_g_	T_g_/°C	T_m_/°C	ΔH/J_g_
PEW1	3.04	38.30	89.59	137.60	179.67	195.95	1.64
PEW2	3.84	39.78	88.68	309.60	184.22	203.16	2.07

^1^ PEW1: pork gelatin, eggshell and 0.08 wt% walnut shell; PEW2: pork gelatin, eggshell and 0.14 wt% walnut shell. All values are mean ± SE. Glass transition temperature (Tg), melting temperature (Tm) and enthalpy (ΔHc) of individual and multilayer films were obtained by differential scanning calorimetry.

## Data Availability

The date are available from the corresponding author.
